# Design of a novel affinity probe using the cell wall-binding domain of a *Listeria monocytogenes* autolysin for pathogen detection

**DOI:** 10.1128/spectrum.05356-22

**Published:** 2023-10-05

**Authors:** Min Lin, Hanhong Dan

**Affiliations:** 1 Ottawa Laboratory (Fallowfield), Canadian Food Inspection Agency, Ottawa, Ontario, Canada; 2 Department of Biochemistry Microbiology and Immunology, University of Ottawa, Ottawa, Ontario, Canada; Pennsylvania State University, State College, Pennsylvania, USA

**Keywords:** *Listeria monocytogenes*, autolysin, GW protein, cell wall-binding domain, lipoteichoic acid, pathogen detection, bacterial capture

## Abstract

Rapid identification of foods and food-processing environments contaminated with *Listeria monocytogenes* is crucial for controlling human listeriosis, a leading cause of fatality related to foodborne illnesses. The *L. monocytogenes* autolysin IspC anchors to the bacterial surface non-covalently via its C-terminal cell wall-binding domain (CWBD), which contains conserved Gly-Trp (GW) dipeptides. This study explored the binding property of CWBD_IspC_ to design an affinity probe for detecting *L. monocytogenes*. Immunogold electron microscopy analysis of a ∆*ispC* mutant and its wild type (WT) revealed that exogenously added CWBD_IspC_ bound to surface ligands that were evenly distributed and remained mostly unoccupied in the wild type. CWBD_IspC_ detected, via recognizing the cell wall component lipoteichoic acid, *L. monocytogenes* strains primarily belonging to the serotype 4 group, as well as *Listeria innocua*, and *Listeria welshimeri* but did not detect three other *Listeria* spp. (*Listeria seeligeri*, *Listeria ivanovii,* and *Listeria grayi*) nor any non-*Listeria* spp. tested. CWBD_IspC_, when tagged with the enhanced green fluorescent protein, was capable of detecting *L. monocytogenes* by fluorescence microscopy, a colony lift filter assay, and a microtube binding assay. CWBD_IspC_, when covalently attached to magnetic beads, showed the ability to separate *L. monocytogenes* cells with a maximum capture rate of 90 to nearly 100% from 1 × 10^3^ to 1 × 10^5^ target cells. This study demonstrated for the first time that the CWBD derived from a GW surface protein IspC is a novel antibody alternative for the rapid detection and isolation of *L. monocytogenes* despite binding to two other *Listeria* species.

**IMPORTANCE:**

Human listeriosis is caused by consuming foods contaminated with the bacterial pathogen *Listeria monocytogenes*, leading to the development of a severe and life-threatening foodborne illness. Detection of *L. monocytogenes* present in food and food processing environments is crucial for preventing *Listeria* infection. The *L. monocytogenes* peptidoglycan hydrolase IspC anchors non-covalently to the bacterial surface through its C-terminal cell wall-binding domain (CWBD), CWBD_IspC_. This study explored the surface binding property of CWBD_IspC_ to design, construct, characterize, and assess an affinity molecular probe for detecting *L. monocytogenes*. CWBD_IspC_ recognized a cell wall ligand lipoteichoic acid that remains evenly displayed and mostly unoccupied on the bacterial surface for interaction with the exogenously added CWBD_IspC_. CWBD_IspC_, when fused to the enhanced green fluorescent protein reporter or covalently conjugated onto magnetic beads, exhibited the functionality as an antibody alternative for rapid detection and efficient separation of the pathogen.

## INTRODUCTION

*Listeria monocytogenes*, a widespread Gram-positive bacterium in natural environments, can cause a rare but serious foodborne illness referred to as listeriosis in humans, particularly susceptible individuals including pregnant women, newborns, older adults, and immunocompromised people, through consuming contaminated foods (1). Strains of this pathogenic microorganism are genetically diverse and are serologically differentiated into at least 13 serotypes and phylogenetically grouped into four distinct lineages (I, II, III, and IV) (2). Serotypes 1/2a, 1/2b, and 4b represent at least 95% of *L. monocytogenes* strains responsible for human cases of listeriosis (3). Rapid identification of food sources and food processing environments contaminated with *L. monocytogenes* is crucial for controlling the spread of the pathogen but remains challenging due to a number of interfering factors including the low level of the pathogen present, the complex food matrices, background microflora, the high genetic diversity of *L. monocytogenes* strains, and lack of suitable affinity probes for highly efficient detection of a wide spectrum of *L. monocytogenes* serotypes. In the past, studies have focused on the development and use of monoclonal antibodies (MAbs) to *L. monocytogenes* antigens for pathogen detection and isolation with some promising results (4 – 17). Some significant drawbacks of MAbs for the detection of *L. monocytogenes* are the use of animals in antibody production and the tedious, time-consuming process of MAb screening and identification despite the mature hybridoma technology used (11, 13, 15 – 17). Specific molecular probes with high affinity for the cell surface ligands common to a wide range of *L. monocytogenes* strains are always desirable and sought as new alternatives to antibodies for the use in the development of rapid detection methods for *L. monocytogenes* present in food, environmental, or clinical samples. One of the mechanisms employed by Gram-positive bacteria to anchor proteins to the cell surface is through the non-covalent interaction of cell wall-binding domains (CWBDs) with the cell wall components (18 – 21). This study aims to design and evaluate a CWBD-based affinity probe for such intended applications.

In previous studies, we identified and characterized an immunogenic surface protein IspC as one of the targets of humoral immune response to *L. monocytogenes* infection (22). IspC is a surface peptidoglycan hydrolase (autolysin) composed of an N-terminal N-acetylglucosaminidase activity domain (aa 1–197) and a C-terminal CWBD (aa 198–774) here referred to as CWBD_IspC_ (23, 24). It also plays a role in *L. monocytogenes* virulence (25). Discovery of the CWBD_IspC_ involved in anchoring the IspC protein to the cell surface through non-covalent interaction with the cell wall (23) has prompted us herein to explore this binding property to design novel strategies for the detection and isolation of *L. monocytogenes*. In *L. monocytogenes*, the internalin B (InlB) protein was first demonstrated to contain a C-terminal CWBD_InlB_ (aa 399–630) composed of 3 Gly-Trp (GW) modules (motifs) each having a ~80 aa sequence and containing a GW dipeptide (19, 26, 27). This type of GW-containing CWBD, interacting with the cell wall component lipoteichoic acid (LTA), functions as one of the non-covalent cell surface anchoring mechanisms in Gram-positive bacteria (18, 19, 27). However, contrasting results from a recent study show that galactosylated WTA is required and sufficient for the display of InlB on the cell surface of *L. monocytogenes* serotype 4b (28). The polyribitol phosphate-based wall teichoic acid (WTA) and the polyglycerol phosphate-based LTA are two different structural classes of surface exposed glycopolymers in the *Listeria* cell wall (28). WTA is covalently linked to the peptidoglycan, whereas LTA is anchored to the cytoplasmic membrane via a lipid moiety. IspC, together with other proteins such as InlB, Ami, and Auto, belongs to a class of cell wall-anchored, GW module-containing surface proteins in *L. monocytogenes* (18, 19, 21, 23). A CWBD composed of a higher number of GW modules renders a stronger attachment of the protein to the cell wall, as illustrated by the eight GW module-containing Ami in comparison to the three GW module-containing InlB (19, 26). *Staphylococcus* species are another Gram-positive bacteria that use a similar cell wall-anchoring mechanism for the surface display of GW module-containing proteins (18 – 20) including several surface autolysins like AtlC from *Staphylococcus caprae* (29), AtlE from *Staphylococcus epidermidis* (30), and Aas from *Staphylococcus saprophyticus* (31).

In some Gram-positive bacteria, including *Streptococcus pneumoniae* and *Clostridium acctobutylicum*, non-covalent anchoring of surface proteins is accomplished by the binding of the C-terminal domains to choline residues in the WTA or LTA (19, 20). The CWBD of a *S. pneumoniae* autolytic amidase LytA is a C-terminal domain, composed of serval imperfect repeats of ~20 aa, which specifically recognizes choline residues (32). PspA, an important virulence factor and vaccine candidate anchored to the cell surface in *S. pneumoniae*, possessing a C-terminal proline-rich CWBD made up of 10 highly conserved 20-aa repeats (20), is another well-characterized choline-binding protein. C-terminal CWBDs are also present in several *L. monocytogenes* bacteriophage peptidoglycan hydrolases (endolysins) (33, 34), thus bringing their N-terminal enzymatic domains close to the substrates by binding to the cell wall ligands. In contrast to the CWBDs of *L. monocytogenes* GW surface proteins such as IspC, InlB, Ami, and Auto, the C-terminal CWBDs of *Listeria* phage endolysins contain no GW modules or other sequence repeats, bind the cell wall devoid of LTA or proteins, and exhibit the specificity with a high binding affinity for the cell wall carbohydrates (33). Uncovering a variety of CWBDs in the surface proteins of Gram-positive pathogenic bacteria has left the door wide open to the possibility of developing CWBD-based innovative tools for rapid pathogen detection. The CWBDs derived from *L. monocytogenes* phage endolysins have found applications in the detection and isolation of the pathogen from food culture enrichment samples using magnetic separation (35 – 37). To date, no study has been conducted to explore the use of CWBDs derived from a class of *L. monocytogenes* GW surface proteins in bacterial detection and isolation. The C-terminal CWBD present in the IspC protein of *L. monocytogenes* strain LI0521 (serotype 4b) contains seven GW modules (23) and is hypothesized to confer strong non-covalent attachment to the cell surface, thereby serving as a new alternative to MAbs for the pathogen detection. The present investigation focused on the design, characterization, and evaluation of a CWBD_IspC_-based affinity probe, either fused with the enhanced green fluorescent protein (EGFP) reporter or covalently conjugated onto magnetic beads (MB), for the rapid detection of *L. monocytogenes* by using fluorescence microscopy (FM), microtube binding assay, or colony lift filter assay and efficient isolation of pathogenic bacteria from culture enrichment samples by using magnetic separation. Here, we demonstrate that CWBD_IspC_, the first example of a CWBD from a class of GW surface proteins, is suitable for the detection and isolation of important *L. monocytogenes* serotypes such as 4b with high efficiency despite research efforts still being needed to identify GW module-containing CWBDs that specifically recognize all serotypes of *L. monocytogenes*.

## MATERIALS AND METHODS

### Culture of bacteria

*Listeria monocytogenes* strains and non-*L*. *monocytogenes* bacterial species (Table 1) were cultured in brain heart infusion (BHI) broth with gentle shaking or agar medium (Becton Dickinson, Sparks, MD, USA) at 37°C for 16–18 h for use in the study. *L. monocytogenes* strain LI0521 (serotype 4b), from which the CWBD_IspC_ coding sequence was derived, was used as a control strain for the binding of CWBD_IspC_ to bacterial cells. *E. coli* strains, DH5α and BL21(DE3)/pLysS used for molecular cloning and protein expression experiments, respectively, were grown in Luria–Bertani (LB) medium.

**TABLE 1:** Examination of the EGFP-CWBD_IspC_ fusion protein binding to the cell surface of various bacterial species by FM

Species	Serotype^*a*^	Strain^*b*^	Origin	FM result^*c*^
*L. monocytogenes*	1/2a	LI0527	Unknown	−
	1/2a	OLF09049	Environment	−
	1/2a	HPB5327	Animal	−
	1/2a	HPB6036	Food	−
	1/2a	HPB6095	Food	−
	1/2b	HPB4857	Animal	−
	1/2b	OLF09060	Environment	−
	1/2c	HPB5121	Food	−
	1/2c	HPB2972	Unknown	−
	3a	HPB5665	Food	−
	3a	HPB3058	Food	−
	3b	HPB1031	Food	−
	3b	HPB4909	Food	−
	3c	HPB61	Unknown	−
	4a	HPB3501	Clinical	+
	4a	HPB5041	Animal	+
	4b	HPB1848	Unknown	+
	4b	HPB3449	Food	+
	4b	HPB5251	Animal	+
	4b	HPB5364	Animal	+
	4b	HPB5816	Food	+
	4b	HPB5906	Food	+
	4b	HPB6024	Food	+
	4b	HPB6092	Food	+
	4b	LI0521	Clinical	+
	4ab	HPB1265	Clinical	+
	4ab	HPB5058	Animal	+
	4ab	HPB520	Environment	+
	4c	HPB5248	Animal	+
	4c	HPB4497	Unknown	+
	4c	HPB4706	Animal	+
	4d	HPB4534	Clinical	−
	4e	HPB18	Unknown	−
	4e	HPB1861	Food	−
*L. innocua*	NA	CFIA	Unknown	+
	6a	CLIP 11262	Food	−
	6a	ATCC 33090 (PHB583)	Cow (brain)	+
*L. seeligeri*	NA	HPB24	Unknown	−
*L. ivanovii*	5	ATCC 19119 (PHB28)	Sheep	±
*L. welshimeri*	NA	HPB92	Food	+
*L. grayi*	NA	ATCC 19120 (PHB29)	Human (feces)	−
*Lactobacillus casei*	NA	ATCC 334	Food	−
*Bacillus cereus*	NA	B3-37	Unknown	−
*Bacillus subtilis*	NA	ATCC 19659	Unknown	−
*Enterococcus faecalis*	NA	ATCC 19433	Unknown	−
*Staphylococcus aureus*	NA	ATCC 25923	Clinical	±
*Streptococcus dysgalactiae* subsp. *equisimilis*	NA	G148	Unknown	−
	NA	BSL2	Human	−
*Enterobacter cloacae* subsp. *cloacae*	NA	ATCC 13047	Spinal fluid	−
*Enterobacter sakazaki*	NA	Strain 2871	Unknown	−
*E. coli (non-pathogenic*)	NA	ATCC 25922	Clinical	−
*E. coli EHEC*	O157:H7	ATCC 43889	Human (feces)	−
	O157:H7	Strain 871215	Unknown	−
	O111:NM	Strain 00-4748	Human	−
	O111	Strain 1950	Unknown	−
	O26:H11	Strain 02-6737	Unknown	−
	O121:H19	Strain 03-2832	Human	−
	O145:HNM	Strain 03-4699	Human	−
	O103:H2	Strain 04-2446	Human	−
	O245:H2	Strain 5645	Unknown	−
*Pseudomonas aeruginosa*	NA	ATCC 27853	Human (blood)	−
*Alcaligenes faecalis* subsp*. faecalis*	NA	ATCC 35655	Unknown	−
*Aeromonas hydrophila*	NA	ATCC 35654	Unknown	−
*Campylobacter jejuni*	NA	ATCC 700819	Human (feces)	−
*Campylobacter fetus* subsp*. fetus*	NA	ATCC 27374	Sheep fetus (brain)	−
*Salmonella enterica* subsp*. enterica*	Typhimurium	ATCC 14028	Chicken	−
	Berta	ATCC 8392	Unknown	−

^*a*^ NA, not available.

^*b*^ Bacterial strains selected for the study are those of the 13 recognized *L. monocytogenes *serotypes, other *Listeria* spp., foodborne bacterial pathogens, Gram-positive and -negative bacteria frequently found in foods and the environment. These strains were collected by our laboratory over the years.

^*c*^ Positive results (+) indicate fluorescence emitted from bacterial cells that have been complexed with EGFP-CWBD_IspC_; negative results (−) indicate no or negligible fluorescence signal emitted from bacterial cells following the reaction with EGFP-CWBD_IspC_. Negative results (−) were also observed with purified EGFP. Inconclusive results are indicated by (±). The results shown here are reproducible with three biological replicates.

### Generation of expression constructs

All DNA manipulations were essentially performed according to the established procedures (38). The genomic DNA of *L. monocytogenes* serotype 4b strain LI0521 was prepared from a 10 mL overnight BHI culture by using DNAZol (Invitrogen, Burlington, ON, Canada) as per the manufacturer’s instructions (39). All of the oligonucleotide primers used are described in Table 2. To create an expression construct for the CWBD (aa 198-774) of IspC (23), the coding sequence was derived from the genomic DNA by PCR with Platinum Pfx DNA polymerase (Invitrogen) and the primer pair P965 and P964. The PCR product was purified following agarose gel electrophoresis procedure using a QIAquick gel extraction kit (Qiagen, Mississauga, ON, Canada) and then cloned into the expression vector pLIC-NHis by the ligation-independent cloning protocol (40), resulting in a construct pCWBD_IspC_ for the production of the recombinant CWBD_IspC_ with an N-terminal 6× His tag.

**TABLE 2:** Oligonucleotide primers used in the study

Primer	Sequence^*a*^ (5′–3′)	Target protein coding sequence
P965	5′ CACCATCATCATCATCACGGTGCCAAATATGATGTTTTATACG 3′	CWBD_IspC_
P964	5′ TTATCCACTTCCACGCTATTTAACGTTTGTAAAAGCTC 3′	
P1086	5′ CACCATCATCATCATCACGGTATGGTGAGCAAGGGCGAG 3′	EGFP
P1089 P1203	5′ **CATATTTGGC**CTTGTACAGCTCGTCCATGC 3′ 5′ **AGCGTGTTAA**CTTGTACAGCTCGTCCATGC 3′	
P1088	5′ GCTGTACAAGGCCAAATATGATGTTTTATACGA 3′	CWBD_IspC_
P1087	5′ TTATCCACTTCCACGCTATTTAACGTTTGTAAAAGCTCG 3′	
P1202	5′ **GCTGTACAAG**TTAACACGCTATGTTAAATATATTC 3′	CWBD_InlB_
P1201	5′ TTATCCACTTCCACGTCATTTCTGTGCCCTTAAATTA 3′	

^*a*^ Underlined sequences are additional nucleotides included at the 5′ end of each primer to meet the requirements for cloning into the pLIC-NHis vector; bold sequences added at the 5′ end are for generating the sequences coding for the fusion proteins EGFP-CWBD_IspC_ or EGFP-CWBD_InlB_ by using overlap extension PCR.

To create an expression construct for CWBD_IspC_ tagged with EGFP at the N-terminus, the coding sequence for the EGFP-CWBD_IspC_ fusion was synthesized using a two-step PCR strategy (23). Briefly, the EGFP coding sequence (omitting the stop codon) was PCR amplified from the plasmid pEGFP-N1 (Clontech Laboratories, Santa Clara, CA, USA) using Platinum Pfx DNA polymerase and the primers P1086 and P1089, and the CWBD_IspC_ coding sequence derived from the *L. monocytogenes* LI0521 genomic DNA by PCR using the primers P1088 and P1087. Then, the two PCR products were spliced by overlap extension PCR with the primers P1086 and P1087. As described above, the fused DNA fragment was cloned into pLIC-NHis to produce the recombinant plasmid pEGFP-CWBD_IspC_. For the production of the EGFP-CWBD_InlB_ fusion protein, a similar PCR cloning strategy was employed to create the recombinant plasmid pEGFP-CWBD_InlB_ using the primers P1086 and P1203 for the EGFP coding sequence, P1202 and P1201 for the coding sequence of CWBD_InlB_, the CWBD of InlB (aa399-630), and P1086 and P1201 for the EGFP-CWBD_InlB_ fusion protein coding sequence.

All of the recombinant plasmids were propagated in *E. coli* DH5α and verified to contain the correct inserts by colony PCR as described previously (22) with a gene-specific primer (Table 2) and the T7 terminator primer (5′-GCTAGTTATTGCTCAGCGG-3′) followed by Sanger DNA sequencing at the sequencing facility, McGill University (Montreal, QC, Canada).

### Expression and purification of recombinant proteins

*E. coli* BL21(DE3)/pLysS harboring a recombinant plasmid (pCWBD_IspC_ or pEGFP-CWBD_IspC_) was used to express the encoded recombinant protein in LB broth culture as described (41). After growth to an OD at 600 nm of about 0.6 in 2–4 L of LB broth containing 50 µg/mL kanamycin, *E. coli* cells were induced by adding 1 mM isopropyl-β-d-thiogalactopyranoside (final concentration) to express the target protein at 37°C for 3 h and then at room temperature (RT) for 1 h followed by overnight incubation at 4°C. The cells were harvested by centrifugation at 16,900 × *g* at 4°C for 20 min and frozen at −20°C until use. For protein purification, the cell pellets were resuspended in phosphate-buffered saline (PBS, pH 7.2) containing 1 mM phenylmethylsulfonyl fluoride and lysed by passing through a French press twice at 1,500 lb/in^2^. The cell homogenates were spun at 27,000 × *g* for 25 min at 4°C to collect the supernatant containing the soluble recombinant protein, which was purified by affinity liquid chromatography on a column (1 by 1.5 cm) of Ni-nitrilotriacetic acid superflow (Qiagen, Santa Clarita, CA. USA) as described (41). The concentration of purified proteins was determined by using the Bradford method (42) with bovine serum albumin (BSA) as a standard.

### Immunogold labeling for electron microscopy

*L. monocytogenes* serotype 4b strain LI0521 and its ∆*ispC* mutant created in a previous study (25) were used to investigate the attachment of the recombinant CWBD_ispC_ using the immunogold electron microscopy method described previously (11), together with a MAb M2773 specific for the CWBD of IspC (13, 43).

### Fluorescence microscopy

The binding of the EGFP-CWBD_ispC_ fusion protein to the cell surface of *L. monocytogenes* or other bacterial cells was examined by fluorescence microscopy on an Olympus BX60 fluorescence microscope essentially as described (15). Briefly, approximately 2 × 10^8^ bacterial cells from an overnight BHI culture were harvested by centrifugation, washed twice with PBS, and then stained with 800 nM EGFP-CWBD_ispC_ in PBS containing 0.1% (vol/vol) Tween-20 (polyethylene glycol sorbitan monolaurate) (PBS-T) at room temperature for 5 min. Cells were collected by centrifugation at 13,200 × *g* for 60 s, washed twice with 500 µL of PBS-T, and resuspended in 50 µL of PBS-T. Ten microliter of cell suspension was placed on a glass slide and viewed at 100× magnification on the microscope with an excitation 490 nm/emission 510 nm filter set. Fluorescence images of bacterial cells complexed with EGFP-CWBD_ispC_ were captured with a charge-coupled device camera.

### Microtube binding assay with EGFP-CWBD_IspC_

The microtube binding assay was performed by collecting approximately 2 × 10^8^ cells of *L. monocytogenes* or other bacterial cells from an overnight BHI culture into a microtube followed by centrifugation at 13,200 × *g* for 1 min, washing twice with PBS, and then staining with 800 nM EGFP-CWBD_ispC_ in PBS-T at room temperature for 5 min. Stained cells were washed twice with 500 μL of PBS-T, pelleted by centrifugation, and then macroscopically imaged under a GFP-MDS-96/BN excitation stand equipped with eight light sources of 96 ultra-bright blue LEDs (BLS Ltd., Budapest, Hungary). The fluorescent images were captured with a Canon digital camera (5D Mark ii) through an FHS/EF-3GY1 filter (BLS Ltd.) attached to the camera lens.

### Binding of CWBD_ispC_ to proteinase K-treated *L. monocytogenes* cells

Cells of *L. monocytogenes* strain LI0521 were harvested from a 10-mL aliquot of overnight BHI broth culture, resuspended in 100 µL of PBS, and digested with 100 µL of proteinase K (20 mg/mL) at 50°C for 30 min. After the treatments, cells were washed extensively in PBS and stained with 800 nM EGFP-CWBD_IspC_ followed by detection with the microtube binding assay described above.

### Colony lift filter assay with EGFP-CWBD_IspC_

Colonies of *L. monocytogenes* cells grown on BHI agar medium or BBL CHROMagar *Listeria* medium (BD Biosciences, Mississauga, ON, Canada) were lifted onto an Optitran nitrocellulose membrane (Schleicher & Schuell, NH, USA). The membrane was air dried, incubated with 5% (wt/vol) BSA in PBS for 60 min at RT, rinsed twice with PBS-T, and then probed with 800 nM EGFP- CWBD_IspC_ in PBS-T for 5 min. After washing twice with PBS-T, the membrane was imaged under a GFP-MDS-96/BN excitation stand (BLS Ltd.) using a Canon digital camera (5D Mark ii) through an FHS/EF-3GY1GFP filter (BLS Ltd.).

### Competitive binding analysis

The CWBD_IspC_ protein was analyzed for the ability to compete with CWBD_InlB_ for binding to the cell wall LTA in *L. monocytogenes* strain LI0521 by forming a complex of bacterial cells with CWBD_IspC_ followed by fluorescent staining with the EGFP-CWBD_InlB_ fusion protein. Briefly, about 2 × 10^8^ formalin-killed cells were incubated with CWBD_IspC_ (0–5.2 µM) in PBS at RT for 1 h with gentle rotation, washed twice with PBS-T, stained with 3.2 µM EGFP-CWBD_InlB_ in PBS for 1 h, and again washed twice with PBS-T. Fluorescence signals emitted from the surface of stained cells were detected by fluorescence microscopy as above. Alternatively, macroscopic imaging for the stained cell pellets in a microtube was performed under a GFP-MDS-96/BN excitation stand (BLS) as described above.

### Conjugation of the CWBD_IspC_ protein to magnetic beads

Covalent immobilization of the recombinant CWBD_IspC_ to M-270 Epoxy Dynabeads (Dynal, Oslo, Norway) was performed as per the manufacturer’s instructions. Lyophilized beads were suspended in diethyleneglycol-dimethyl ether to a final concentration of 30 mg/mL. A 270 µL aliquot of bead suspension was washed twice each with 800 µL of PBS and then resuspended in 100 µL of PBS. The CWBD_IspC_ protein of various quantities (0, 5, 20, 25, 50, 150, 200, 250, 300, 400, and 500 µg) each in 100 µL of PBS was mixed with 100 µL of bead suspension in PBS, and 100 µL of 3 M (NH_4_)_2_SO_4_ (pH 7.4). The mixtures were incubated in a rotating mixer at 10 rpm for 16 h at 4°C, and then for 6 h at ambient temperature (22°C). Residual epoxy groups on beads were blocked by washing the beads four times with PBS containing 0.1% (wt/vol) BSA. CWBD-conjugated magnetic beads, at a concentration of ~2.0 × 10^9^ beads/mL in PBS containing 0.1% (wt/vol) BSA and 0.02% NaN_3_, were stored at 4°C until use.

### Capture of *L. monocytogenes* with CWBD-conjugated magnetic beads

The ability of CWBD_IspC_ to capture *L. monocytogenes* cells from BHI broth culture samples was assessed with CWBD_IspC_-conjugated magnetic beads in three independent experiments. CWBD_IspC_-coated beads (4.0 × 10^7^ beads in 20 µL) were mixed with 1 mL of bacterial cells (10^3^, 10^4^, or 10^5^ cells/ml) prepared by diluting an overnight BHI culture in PBS. The mixtures were rotated at 10 rpm for 45 min at 4°C. After magnetic separation, beads were washed with 800 µL of PBST for 15 min at room temperature, resuspended in 450 µL of PBST, and vortexed vigorously for 1 min. Three 100 µL aliquots from each bead captured cell suspension were plated, respectively, on BHI agar media, incubated at 37°C for 20–24 h, and examined for the number of colony-forming units (CFUs). The rate of bacterial recovery was estimated by calculating the ratio of the input cell number to the number of CFUs based on the assumption that each CFU on the BHI agar plate is derived from a free single bacterial cell in the captured cell suspension. For comparison, a *Listeria* Capture Kit for Plate (Hyglos GmbH, Bernried, Germany) was used in parallel to capture *L. monocytogenes* cells according to the manufacturer’s instructions.

## RESULTS

### Even distribution of the surface ligand recognized by CWBD_IspC_

**Fig 1:**
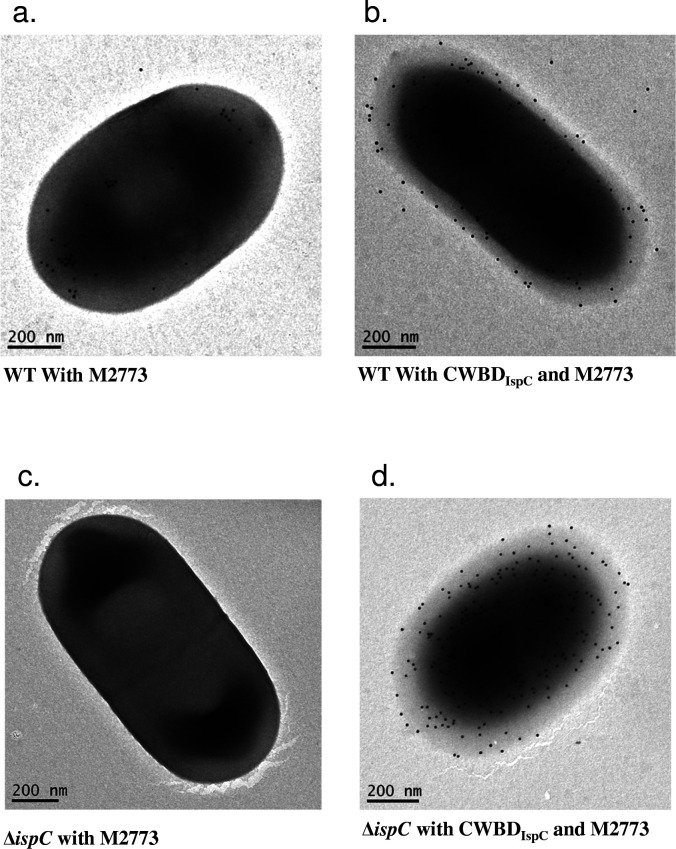
Immunogold labeling of CWBD_IspC_-decorated *L. monocytogenes* cells for scanning electron microscopy. The WT or Δ*ispC* mutant cells of *L. monocytogenes* strain LI0521 serotype 4b were decorated with the recombinant CWBD_IspC_ at 800 nM and then immunogold labeled (black dots in the cell images) with a MAb M2773 against CWBD_IspC_. Bacterial cells without CWBD_IspC_ decoration were used as the control for comparison. (**a**) WT cells probed with M2773; (**b**) WT cells probed with CWBD_IspC_ followed by M2773; (**c**) mutant cells probed with M2773; and (**d**) mutant cells probed with CWBD_IspC_ followed by M2773. The figure depicts one representative of at least two independent experiments that yield reproducible results.

Immunogold labeling with a CWBD_IspC_-specific MAb M2773 revealed that IspC expressed in *L. monocytogenes* was targeted to the cell surface through CWBD_IspC_ interacting with the cell wall ligand (Fig. 1a). IspC localized only to certain regions of the cell surface, primarily the polar regions. Probing *L. monocytogenes* cells with CWBD_IspC_ followed by immunogold labeling with M2773 showed that the exogenously added CWBD_IspC_ decorated the cell surface evenly (Fig. 1b), indicating that only a small number of the ligands formed a complex with IspC expressed in the bacterial cells and the majority of ligands were freely available for interaction with CWBD_IspC_. As expected, immunogold labeling of the *ispC* deletion (∆*ispC*) mutant with M2773 showed no gold particles on the cell surface (Fig. 1c) but displayed an even distribution of gold particles on the surface of the mutant cells pre-probed with CWBD_IspC_ (Fig. 1d). The results of the immunogold labeling experiments demonstrate that ligands serving as the binding sites for CWBD_IspC_ are evenly distributed on the cell surface.

### Non-protein nature of the cell surface ligand involved in binding CWBD_IspC_

To understand the molecular nature of the surface ligand non-covalently bound with CWBD_IspC_, live *L. monocytogenes* that had been treated with proteinase K to digest surface proteins were probed with EGFP-CWBD_IspC_. Fluorescence microscopy showed that protease-treated cells did not appear to alter the binding of the CWBD_IspC_ protein to the cell surface, compared to the untreated control (data not shown). This result indicated that the cell surface component functioning as the binding ligand of CWBD_IspC_ was of a non-protein nature.

### Inhibition of CWBD_InlB_ binding to the cell wall by CWBD_IspC_

**Fig 2:**
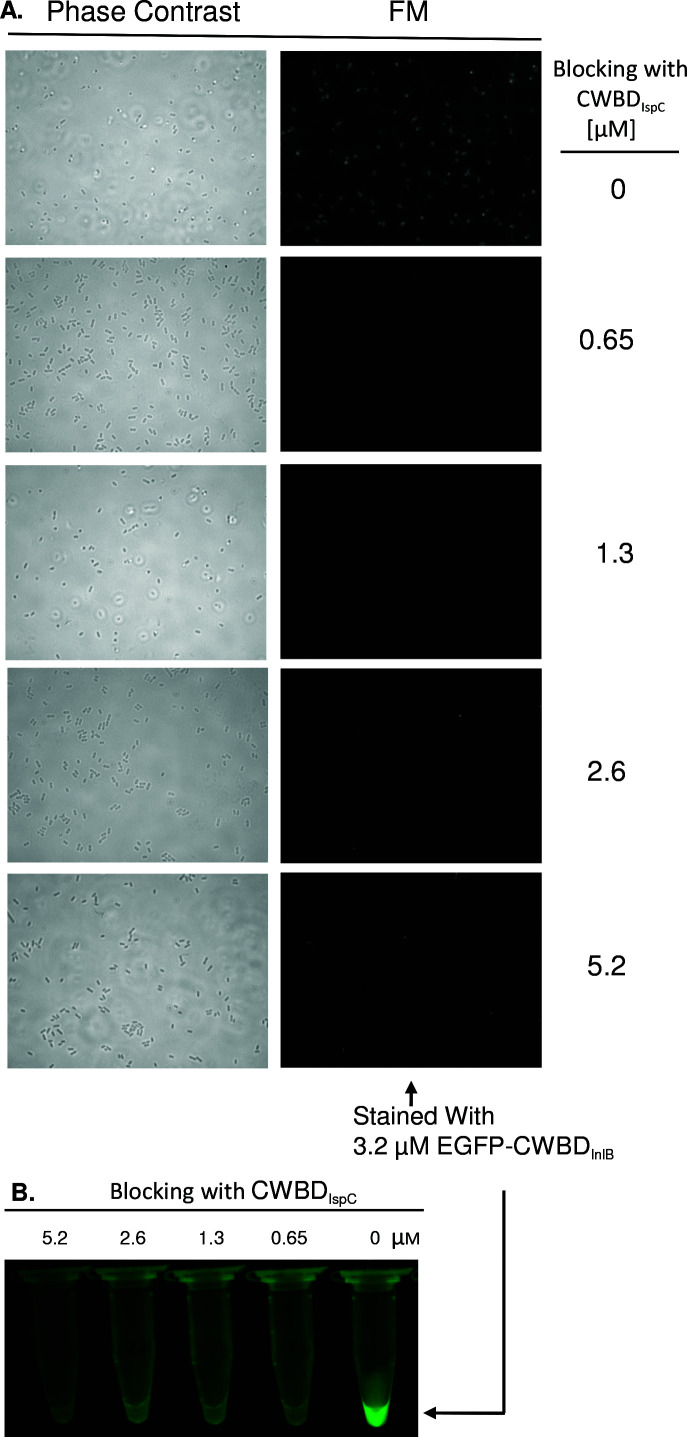
Inhibition of CWBD_InlB_ from binding to the cell surface of *L. monocytogenes* by CWBD_IspC_. Cells of *L. monocytogenes* strain LI0521 serotype 4b were bound with CWBD_IspC_ at various concentrations, stained with 3.2 µM EGFP-CWBD_InlB_, and then detected by fluorescence microscopy (**A**, right panel) in comparison to corresponding phase-contrast microscopy images (**A**, left panel) or by a microtube binding assay under excitation of ultra-bright blue LEDs (**B**). The results obtained with at least two independent experiments are reproducible and exemplified here with one representative of the experiments.

The InlB protein is known to be anchored to the cell surface via the non-covalent interaction of CWBD_InlB_, the C-terminal region (aa 399–630) composed of three GW modules, with the cell wall component LTA (26, 27). To demonstrate that CWBD_IspC_ recognizes the same cell wall component, the CWBD_IspC_ protein at various concentrations was tested for the ability to block the binding of the EGFP-tagged CWBD_InlB_ to *L. monocytogenes*. Fluorescence microscopy revealed that live *L. monocytogenes* that had been pre-decorated with CWBD_IspC_ showed a reduction in fluorescence signals to almost none as the concentration of exogenously added CWBD_IspC_ was increased, compared to the untreated cells (Fig. 2A). Consistent results were also obtained with the microtube binding assay (Fig. 2B), showing that decoration of live *L. monocytogenes* by EGFP-CWBD_InlB_ was blocked near completely by pre-treatment of bacterial cells with increasing concentrations of CWBD_IspC._ The EGFP alone showed no effect on the cell decoration with the EGFP-CWBD_InlB_ (data not shown) in both fluorescence microscopy and microtube binding experiments.

### Specific recognition of the *Listeria* cell wall by CWBD_IspC_

**Fig 3:**
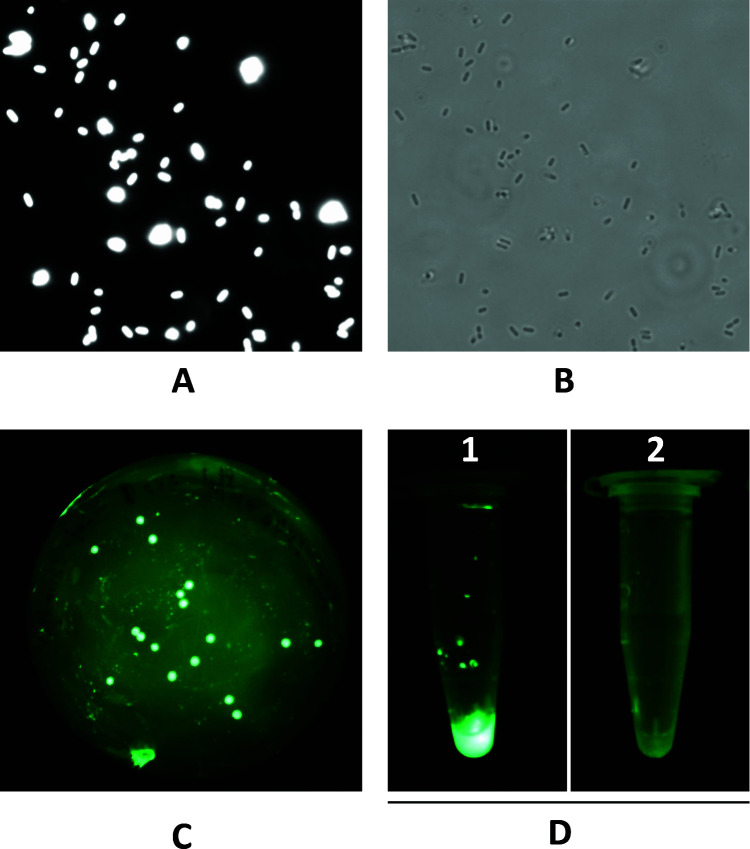
Binding of EGFP-CWBD_IspC_ to the surface of *L. monocytogenes* LI0521 serotype 4b. (**A**) A fluorescence microscopy image shows EGFP-CWBD_IspC_ binds to live *L. monocytogenes* cells. (**B**) A phase-contrast image of bacterial cells in the same field as in panel (A). (**C**) Colony lift filter assay shows a fluorescent image of EGFP-CWBD_IspC_ binding to *L. monocytogenes* colonies. (**D**) A microtube binding assay shows fluorescence emitted from EGFP-CWBD_IspC_ binding to live *L. monocytogenes* cells (tube 1) and to live *E. coil* O157:H7 cells (tube 2). The figure exemplifies one representative of at least two independent experiments that yield reproducible results.

The EGFP-CWBD_IspC_ fusion protein, CWBD_IspC_ tagged with EGFP at the N-terminus, was shown to bind the surface of *L. monocytogenes* LI0521 serotype 4b by using fluorescence microscopy, colony lift filter assay, and microtube binding assay (Fig. 3). In contrast to EGFP-CWBD_IspC_, purified EGFP (control) failed to bind the bacterial cells (no fluorescence detected, data not shown). Fluorescence microscopy analysis of EGFP-CWBD_IspC_ binding to representative strains of the 12 *L*. *monocytogenes* serotypes, 5 other *Listeria* spp., and 15 Gram-negative or -positive non-*Listeria* spp. showed the cell binding specificity of CWBD_IspC_ (Table 1). CWBD_IspC_ was capable of binding *L. monocytogenes*, *L. innocua*, and *L. welshimeri* but not *L. seeligeri*, *L. ivanovii,* and *L. grayi*. For *L. monocytogenes*, EGFP-CWBD_IspC_ was able to bind the strains belonging to serotypes 4a, 4ab, 4b, and 4c but failed to recognize serotypes 1/2a, 1/2b, 1/2c, 3a, 3b, 3c, 4d, and 4e. None of the 15 Gram-negative or -positive non-*Listeria* spp. was recognized by EGFP-CWBD_IspC_.

### Rapid detection of *L. monocytogenes* using CWBD_IspC_-based binding assays

**Fig 4:**
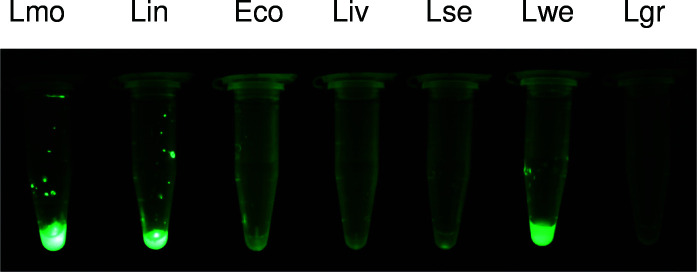
Binding of EGFP-CWBD_IspC_ to six *Listeria* species. A microtube assay shows fluorescence emitted from EGFP-CWBD_IspC_ binding to *Listeria* species, *L. monocytogenes* serotype 4b (Lmo), *L. innocua* (Lin), *L. ivanovii* (Liv), *L. seeligeri* (Lse), *L. welshimeri* (Lwe), and *L. grayi* (Lgr). *E. coli* O157:H7 cells (Eco) serve as negative control. The image captures the reproducible results from one representative of at least two independent experiments.

The application of CWBD_IspC_ in the detection of *L. monocytogenes* was demonstrated in two binding assay formats. The EGFP- CWBD_IspC_ fusion protein was able to detect the target cells from a broth culture in a microfuge tube binding assay (Fig. 4). Alternatively, colonies of *L. monocytogenes* cells grown on an agar medium were detected by EGFP-CWBD_IspC_ after blotting onto a nitrocellulose membrane in a colony lift filter assay (Fig. 3C).

### Efficient *L. monocytogenes* capturing using CWBD_IspC_-based magnetic separation

**Fig 5:**
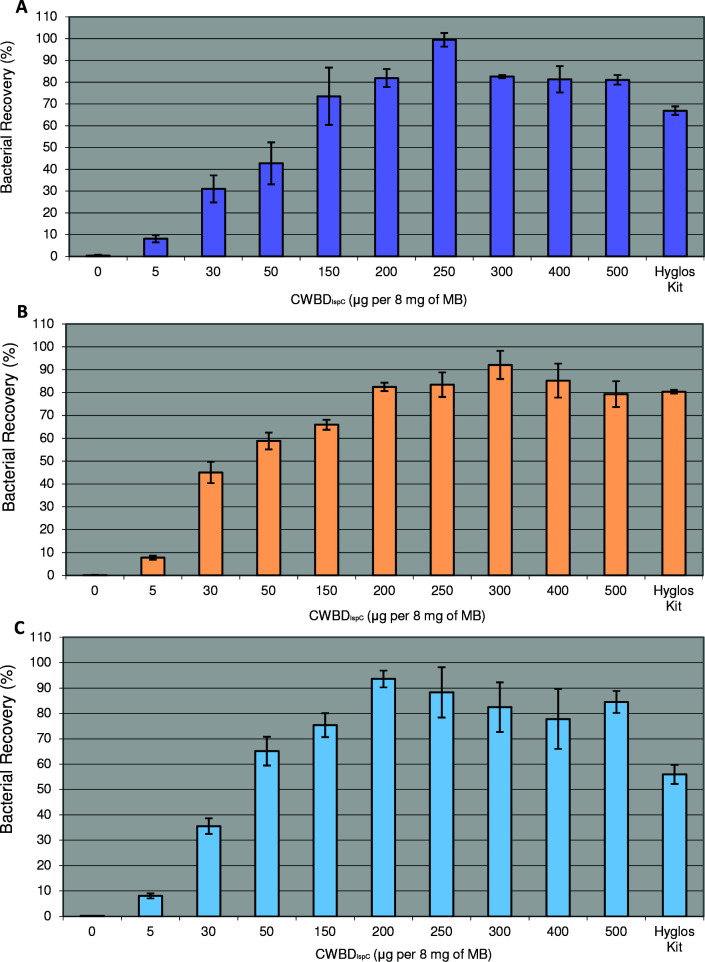
Efficiency of capturing *L. monocytogenes* strain LI0521 serotype 4b by CWBD_IspC_ conjugated to MB. CWBD-coupled magnetic beads were prepared by using various quantities of CWBD (0–500 µg) and a fixed amount of M-270 Epoxy Dynabeads (8.0 mg). Each conjugate preparation (20 µL) was evaluated for the efficacy to capture bacterial cells from 1 mL of samples, prepared from the dilution of a BHI culture in PBS to contain 1 × 10^3^ (**A**), 1 × 10^4^ (**B**), and 1 × 10^5^ (**C**) cells. The data represented by each bar are the mean ± SD calculated from three experimental replicates with three technical replicates. The data obtained in parallel with a commercial *Listeria* capture Kit are shown for comparison (Hyglos Kit). Beads without CWBD_IspC_ conjugation serve as a control for bacterial capture.

Preparations of CWBD_IspC_ conjugated to the surface of M-270 Epoxy Dynabeads at various amounts of the protein to a fixed amount of beads were assessed for their capability to capture *L. monocytogenes* strain LI0521 (serotype 4b) from three input quantities of target cells (1 × 10^3^, 1 × 10^4^, and 1 × 10^5^ cells). In all cases, the beads without CWBD_IspC_ coupling (control) showed approximately 0% cell recovery, while an increase in the amount of the protein used for conjugation with a fixed quantity of beads led to an increase in the capture efficiency with a maximum of 90 to near 100% at a ratio of 200–300 µg CWBD_IspC_ to 8 mg beads (Fig. 5). Continuing to increase the amount of the CWBD_IspC_ protein used in the conjugation after reaching a maximum of capture efficiency showed no improvement in the bacterial capture. At the optimum ratios (wt/wt) of the CWBD_IspC_ protein to magnetic beads, CWBD_IspC_ resulted in a higher capture efficiency than a commercial *Listeria* capture kit (Fig. 5). It is worth noting that CWBD_IspC_ beads can capture at least two out of six *L*. *monocytogenes* LI0521 cells in 1 mL PBS (M. Lin and H. Dan, unpublished data).

## DISCUSSION

This investigation derived the GW module-containing CWBD_IspC_ from the *L. monocytogenes* surface autolysin IspC and demonstrated the suitability of this domain as a new alternative to antibodies for rapid detection of pathogenic bacteria. CWBD_IspC_, via the non-covalent association with the cell wall non-protein component LTA, is responsible for anchoring IspC to the cell surface. As demonstrated here, the construction of an EGFP-tagged CWBD_IspC_ fusion protein allowed direct detection of the target pathogen in various assay configurations such as a fluorescence microscopy-based assay, a microtube binding assay, or a colony lift filter assay. It is reasonably foreseeable that CWBD_IspC_ could be applied in substitution for antibody in the development of other assays for pathogen detection, including flow cytometry assay and immunochromatography assay. These CWBD_IspC_-based detection methods are efficient and fast compared to conventional antibody-based immunological assays for pathogen detection because they do not require secondary antibodies and thus require fewer steps than the immunological assays. CWBD_IspC_, when coated onto magnetic beads for bacterial separation, showed a striking ability to capture *L. monocytogenes* cells from culture samples (strain LI0521 serotype 4b) with a recovery efficiency of up to near 100% (Fig. 5). In addition, recombinant production of the EGFP-CWBD_IspC_ chimera or the CWBD_IspC_ *in vitro* using *E. coli* cells for the diagnostic applications is important and significant because this approach does not involve the use of live animals for antibody development and permits scaling up of production of antibody alternatives.

Anchoring of the endogenously expressed IspC to the cell surface via the non-covalent association of CWBD_IspC_ with its binding ligand (23) necessarily occupies the CWBD_IspC_ binding sites and may decrease the effectiveness of detecting or capturing *L. monocytogenes* cells using exogenously added recombinant CWBD_IspC_. It is crucial to assess the availability of the CWBD_IspC_-binding sites on the cell surface of live *L. monocytogenes* to explore the practical application of CWBD_IspC_ in pathogen detection. Examination of the wild-type and the ∆*ispC* mutant cells of *L. monocytogenes* by electron microscopy with the CWBD_IspC_ protein and an MAb specific for CWBD_IspC_ showed an even distribution of the CWBD_IspC_ binding sites on the entire cell surface, while decoration of the binding sites by the IspC expressed in *L. monocytogenes* primarily clustered within certain surface regions such as the poles and septal regions. This observation implies that the majority of binding ligands are freely available on the surface to serve as targets for detecting *L. monocytogenes* with exogenously expressed CWBD_IspC_. The use of the CWBDs derived from bacteriophage-encoded peptidoglycan hydrolases (endolysins) to capture and separate *L. monocytogenes* from selective enrichment cultures of artificially contaminated food samples was successfully demonstrated in previous studies (35 – 37). Instead of binding WTA or LTA, the CWBDs of bacteriophage endolysins specifically recognize the cell wall carbohydrate component peptidoglycan in *L. monocytogenes,* and WTA spatially limits access of phage endolysins to the peptidoglycan component (33, 44). The CWBD_IspC_, similar to the CWBD_InlB_ in GW module organization, was shown to recognize the membrane-anchored cell wall ligand LTA as it was capable of inhibiting the LTA-binding CWBD derived from InlB from binding to the cell surface of *L. monocytogenes*. Thus, CWBD_IspC_, representing a structural class of CWBD different from the CWBDs of phage endolysins involved in the mechanism of anchoring surface protein, is being explored for the detection of *L. monocytogenes* in the present investigation. Given that teichoic acids are the most abundant cell wall component of *Listeria* existing as two different structural classes of glycopolymers WTA and LTA (28), specific targeting of LTA by a CWBD such as the CWBD_IspC_ of *L. monocytogenes* is expected to have a diagnostic value when CWBD_IspC_ is incorporated into laboratory tests for the pathogen detection and separation.

Tagging the reporter EGFP to CWBD_IspC_ allowed for the visualization of surface decoration of *L. monocytogenes* with CWBD_IspC_ by fluorescence microscopy, as similarly reported for the CWBDs of some bacteriophage endolysins (33). The CWBD of a *Staphylococcus aureus* autolysin when fused with the green fluorescent protein (GFP) was capable of decorating *S. aureus* cells visualized by fluorescence microscopy (45). These observations of the cell surface decoration with an EGFP- or GFP-CWBD fusion protein have created motivation for the development of GFP fusion protein-based assays for the rapid detection of *L. monocytogenes*, as successfully illustrated here with a microtube binding assay and a colony lift filter assay. These assay strategies may be extended to design assays for the rapid detection of any Gram-positive bacterial pathogens using the CWBDs derived from non-covalent cell wall-associated surface proteins encoded by bacterial genomes.

CWBD_IspC_ did not display specificity for any of the non-*Listeria* bacterial species tested, regardless of whether they were Gram-positive or Gram-negative. This observation is consistent with the findings that the CWBDs from *L. monocytogenes* bacteriophage endolysins generally did not bind to Gram-positive or -negative bacteria other than *Listeria* cells (33). One exception to this specificity was that the CWBD of a *L. monocytogenes* bacteriophage endolysin recognized *Bacillus megaterium* (33). In contrast to the CWBD_IspC_, the CWBD derived from a *S. aureus* autolysin, which is composed of three repeated sequences (150 aa each), binds to a wide range of Gram-positive bacteria but not to most Gram-negative bacteria (45). These findings underscore the importance of understanding the bacterial cell-binding specificity of a given CWBD in order to explore its diagnostic value in pathogen detection. The CWBD_IspC_ recognized three out of the six *Listeria* spp. tested (*L. monocytogenes*, *L. innocua*, and *L. welshimeri*), suggesting these three species display a structurally similar binding site within the membrane-bound cell wall component LTA on the surface. The CWBD_IspC_ was specific for strains of *L. monocytogenes*, largely restricted to the serotype 4 group, suggesting that a structural variation in LTA exists among *L. monocytogenes* strains. Strains of *L. monocytogenes*, *L. innocua*, and *L. welshimeri* reactive with CWBD_IspC_ were found to encode protein homologs having near or greater than 90% sequence identity with IspC (43). This suggests that the IspC homolog encoded by the genome of *a Listeria* strain allows to make a prediction about the reactivity of this strain with CWBD_IspC_. *Listeria* strains are classified into various serotypes based on the serological reaction of O-antigenic factors with rabbit antisera (46, 47). Interestingly, all CWBD_IspC_-reactive non-4b strains of *L. monocytogenes* or other *Listeria* spp. such as *L. innocua* and *L. welshimeri* contain the O-antigenic factor VII. Lack of the VII factor in *L. seeligeri*, *L. ivanovii*, and *L. grayi* serotypes appears to correlate with the observation that CWBD_IspC_ did not react with these *Listeria* spp. In spite of the specificity and limitation of CWBD_IspC_, this domain is still a useful affinity probe for the detection of *L. monocytogenes* because the detection of other *Listeria* spp., especially a certain level of *L. innocua*, is a good indicator of possible *L. monocytogenes* contamination or poor sanitation in food processing environments (48 – 50). Nonetheless, CWBD_IspC_ serves as a promising model of GW module-containing CWBDs being explored as a new antibody alternative for the rapid detection of *L. monocytogenes*. It is worth making continuous efforts to screen other non-covalent cell wall-anchored proteins encoded by the genome of *L. monocytogenes* to possibly discover CWBDs specifically recognizing *L. monocytogenes*, given that a number of surface proteins containing GW modules or LysM domains are identified in *L. monocytogenes* (18, 51).

MAbs coated onto magnetic beads via covalent linkages were demonstrated to be successful in capturing and separating *L. monocytogenes* from pure cultures or selective enrichment cultures of artificially contaminated foods; however, low rates of bacterial capture were generally obtained (5, 52). This study showed CWBD_IspC_ as a new antibody alternative for capturing *L. monocytogenes* from pure culture when covalently coupled to magnetic beads. A much higher capture efficiency of 90 to near 100% with CWBD_IspC_ than those (2.0–3.7 %) with MAbs (52) showed a considerable strength of CWBDs for *L. monocytogenes* capture and separation. Similarly, the CWBDs of *L. monocytogenes* bacteriophage endolysins and of a *S. aureus* autolysin were demonstrated to be very efficient in the magnetic bead-based separation of target pathogenic bacteria (35 – 37, 45). Several factors including high binding affinity and high binding site density may have strengthened the ability of CWBD_IspC_ to overcome the limitation of conventional MAbs to achieve an excellent efficiency in the separation and detection of *L. monocytogenes*. The CWBD_IspC_ presumably has a high affinity for the cell surface ligand LTA due to its seven GW modules (23), since the *L. monocytogenes* autolysin Ami containing eight GW modules exhibited a much stronger cell surface attachment in comparison to the three GW module-containing InlB (19, 26, 27, 53). The CWBDs of *L. monocytogenes* bacteriophage endolysins were shown to have high affinity with equilibrium dissociation constant *K_D_* values in the nanomolar range (1/3 × 10^−8^–1/6 × 10^−8^ M) (33), while the CWBD of a *S. aureus* autolysin bound to the cells with a *K_D_* of 1.5 × 10^−8^ M (45). Electron microscopy semi-quantitatively revealed a high density of binding sites for CWBD_IspC_, which is consistent with the observation that a *L. monocytogenes* cell displays 4 × 10^4^–8 × 10^4^ CWBD-binding sites on the cell surface for bacteriophage endolysins (33). Another element that may contribute to the effectiveness of using CWBD_IspC_ as an antibody alternative is that its binding ligand LTA is a major non-protein constituent of the cell wall, in contrast to the surface protein antigens recognized by anti-*L*. *monocytogenes* MAbs, which may be expressed at a low level or down-regulated under certain environmental conditions. To realize the full potential of a CWBD_IspC_-based detection method, it is necessary to assess its application to the detection of target *L. monocytogenes* serotypes in naturally or artificially contaminated food samples.

In conclusion, this study demonstrated the development of CWBD_IspC_, a GW module-containing CWBD derived from a *L. monocytogenes* peptidoglycan hydrolase IspC, as a novel alternative to traditional MAbs for the pathogen detection and isolation. The CWBD_IspC_ exhibited a much higher efficiency in bacterial detection and separation in comparison to MAbs, presumably due to the high affinity of CWBD_IspC_ for bacterial cell recognition, the high density of binding sites on the cell surface, and the specificity for a major non-protein cell wall component LTA. Some limitations are associated with the use of CWBD_IspC_ for *L. monocytogenes* detection as this CWBD recognizes two other *Listeria* species, *L. innocua* and *L. welshimeri*, besides *L. monocytogenes* and binds *L. monocytogenes* strains primarily belonging to the serotype 4 group. Because of these limitations, future research efforts need to be focused on the discovery of novel *L. monocytogenes*-specific CWBDs for diagnostic applications through bioinformatics analysis of the genome sequences of various *L. monocytogenes* strains available in the public databases. In this way, more GW surface proteins and other non-covalent surface-anchored proteins can be identified for intended use.
